# Downregulation of miR-335 exhibited an oncogenic effect via promoting KDM3A/YAP1 networks in clear cell renal cell carcinoma

**DOI:** 10.1038/s41417-021-00335-3

**Published:** 2021-04-23

**Authors:** Wenqiang Zhang, Ruiyu Liu, Lin Zhang, Chao Wang, Ziyan Dong, Jiasheng Feng, Mayao Luo, Yifan Zhang, Zhuofan Xu, Shidong Lv, Qiang Wei

**Affiliations:** Department of Urology, Nanfang Hospital, Southern Medical University, Guangzhou, Guangdong China

**Keywords:** Renal cancer, Cancer genetics

## Abstract

Clear cell renal cell carcinoma (ccRCC) is the most common type of renal cancer affecting many people worldwide. Although the 5-year survival rate is 65% in localized disease, after metastasis, the survival rate is <10%. Emerging evidence has shown that microRNAs (miRNAs) play a crucial regulatory role in the progression of ccRCC. Here, we show that miR-335, an anti-onco-miRNA, is downregulation in tumor tissue and inhibited ccRCC cell proliferation, invasion, and migration. Our studies further identify the H3K9me1/2 histone demethylase KDM3A as a new miR-335-regulated gene. We show that KDM3A is overexpressed in ccRCC, and its upregulation contributes to the carcinogenesis and metastasis of ccRCC. Moreover, with the overexpression of KDM3A, YAP1 was increased and identified as a direct downstream target of KDM3A. Enrichment of KDM3A demethylase on YAP1 promoter was confirmed by CHIP-qPCR and YAP1 was also found involved in the cell growth and metastasis inhibitory of miR-335. Together, our study establishes a new miR-335/KDM3A/YAP1 regulation axis, which provided new insight and potential targeting of the metastasized ccRCC.

## Introduction

Renal cell cancer (RCC) is one of the most common malignancies worldwide. Based on global cancer 2018 statistics, more than 400,000 patients were newly diagnosed, while more than 170,000 deaths were due to RCC each year [1]. Among all types of RCC, clear cell renal cell carcinoma (ccRCC, also called KIRC) is the most common subtype that accounts for 65–70% of all renal cancers [1, 2]. The 5-year survival rate of localized ccRCC is 65%, however, once the tumor has metastasized, the 5-year survival rate is <10% [3]. This occurs because ccRCC is insensitive to chemotherapy and radiation therapy, and surgery remains the primary choice for patients with ccRCC [4]. Currently, due to the understanding of mechanisms involved in the development and progression of ccRCC, the vascular endothelial growth factor tyrosine kinase inhibitor or rapamycin inhibitor was developed and approved to serve as a first-line treatment for ccRCC [5], highlighting the need to explore ccRCC therapies at the molecular level.

MicroRNAs (miRNAs) are epigenetic regulators of gene expression that contribute to multiple cellular processes, including proliferation, cell fate, and differentiation [5]. MiRNAs regulate gene expression in a sequence-specific manner. Following incorporation into the ribonucleoprotein (RNP) complex RISC (RNA induced silencing complex), miRNAs bind mRNA primarily at their 3′UTRs and impair the translation and/or stability of the target mRNA [6, 7]. Recently, the functional correlation between miRNAs and cancer has been widely reported. A large number of miRNAs are found to act as onco-miRNAs or anti-onco-miRNAs in human cancer [6, 7]. In ccRCC, several miRNAs are identified with altered expression. miR-28, miR-185, miR-27, and let-7f-2 are upregulated in tumor tissue from patients with ccRCC [8], while miR-141 and miR-200c are downregulated [9]. Altered expressions of miRNAs are related to poor cancer-specific survival after tumor excision. Thus, miRNAs are a suitable prognostic and predictive marker to determine survival of ccRCC patients [10]. More importantly, research has shown that these miRNAs contribute to the ccRCC phenotype [11]. In one report, increased expression of miR-28–5p could promote chromosome instability by inhibiting mitotic checkpoint protein Mad2 in VHL inactivated ccRCC [12]. The decreased expression of miR-141 could promote ccRCC proliferation and metastasis by controlling EphA2 [13]. These studies emphasize the importance of miRNAs in ccRCC carcinogenesis. However, due to the complexity of the miRNA regulatory network, pathophysiological functions and downstream targets of miRNAs in ccRCC require further investigation.

In the present study, we identified that miR-335, a miRNA significantly downregulated in ccRCC, inhibits proliferation, migration, and invasion in a ccRCC cell line. Moreover, we found that the H3K9me1/2 histone demethylase KDM3A is a novel miR-335 downstream target. KDM3A is overexpressed in ccRCC and may promote tumor growth and metastasis by activating yes-associated protein 1 (YAP1) expression through epigenetic mechanisms, thus establishing a miR-335/KDM3A/YAP1 regulation axis. Taken together, our study reveals a new miRNA-regulated, epigenetic, tumor-promotional pathway in ccRCC, which provides new insight and potential targeting of metastasized ccRCC.

## Materials and methods

### Bioinformatics analysis

The GEO database (https://www.ncbi.nlm.nih.gov/geo/) was used to search and obtain the human ccRCC miRNA expression dataset GSE12105, GSE16441, and GSE37989, and mRNA expression dataset GSE53757. Differential expression of miRNA and mRNA between ccRCC samples and controls were determined using the “limma” package in R language, with | logFC| > 1 and *p* < 0.05 as the threshold. A “pheatmap” package in R language was used to construct a differential miRNA and mRNA expression heatmap. Three microarray differential miRNA datasets were analyzed using Venn maps (http://bioinformatics.psb.ugent.be/webtools/Venn/). starBase (http://starbase.sysu.edu.cn/index.php) was used to predict downstream mRNA targets of miR-335. The first 45 mRNAs that were significantly upregulated in ccRCC mRNA dataset GSE53757 were included in the analysis. Finally, GEPIA (http://gepia.cancer-pku.cn/index.html) database was used to verify target mRNA expression in ccRCC samples.

### Patient enrollment and tissue collection

A total of 62 patients (27 men and 35 women) pathologically diagnosed with ccRCC were selected from Nanfang Hospital between 2018 and 2020. All patients had undergone radical or partial nephrectomy. All tumor tissues were stored in liquid nitrogen. Patients were classified according to the World Health Organization tumor-lymph node-metastasis classification system. This study followed The Helsinki Declaration and was approved by the Ethics Committee of Nanfang Hospital (NFEC-201910-K2). All patients’ clinical information is shown in Table S1.

### Cell culture and transfection

Renal cancer cell lines 786-O, Caki-1, and Caki-2 were purchased from the American Tissue Culture Collection (ATCC). 786-O cells were cultured with RP1640 medium (Hyclone, Beijing, China). Caki-1 and Caki-2 were cultured with McCoy’s 5 A medium (Gibco, Waltham, MA, USA). HK2 cells (kidney proximal tubule epithelial cells) were cultured in DMEM/F-12 medium (Hyclone). 293T cells were purchased from the Chinese Academy of Sciences cell bank and cultured with DMEM medium (Hyclone). All cells were cultured in a medium supplemented with 10% fetal bovine serum (FBS; Hyclone) and 1% penicillin/streptomycin (Invitrogen, Waltham, MA, USA) in a 5% CO_2_ incubator at 37 °C.

miR-335 mimics (UCAAGAGCAAUAACGAAAAAUGU) and NC-mimics (CUACAAAGCAAUAACGGGGGAUG) were synthesized by RiboBio (Guangzhou, Guangdong, China). Overexpression pcDNA3.1 (+) vector was purchased from Invitrogen. KDM3A shRNA (shKDM3A-1: GGAGAAGAUUUUAGAGAUA; shKDM3A-2: UGUCAAAGGUGUUCGAGAA), YAP1 shRNA (shYAP1: GGUCAGAGAUACUUCUUAA), and negative control scramble-shRNA (shNC: AUUUCGACCAAGGGCAGUA) were purchased from (Sigma, St. Louis, MO, USA). shRNA, overexpression plasmid, and miRNA mimics were all transfected using LIPO2000 reagent.

### Antibodies and reagents

KDM3A (ab91252, Abcam, Cambridge, MA, USA), Ki67 (ab16667, Abcam, Cambridge, MA, USA), E-cadherin (CST, #3195, Cell Signaling Technology, Danvers, MA, USA), Vimentin (CST, #5741, Cell Signaling Technology, Danvers, MA, USA), Hippo pathway antibody sampler kit (CST 8579, Cell Signaling Technology, Danvers, MA, USA), including LATS1, MST1, MST2, YAP1, TAZ, and GAPDH (sc-365062, Santa Cruz, CA, USA) were used in this study. H3K9m2 inhibitor Bix01294, UNC0631, Hippo pathway inhibitor verteporfin were purchased from Selleck (Houston, TX, USA).

### mRNA expression determined using qRT-PCR

Cells or tissues were lysed using a Trizol kit (Invitrogen). Total RNA was extracted using the RNeasy Mini Kit (Qiagen, Valencia, CA, USA). cDNA was synthesized using the miRNA First Strand cDNA Synthesis (Tailing Reaction) kit (B532453–0020, Sangon, Shanghai, China). cDNA was made from mRNA detection using a reverse transcription kit (RR047A, Takara, Kyoto, Japan). Ultraviolet-visible spectrophotometry (ND-1000, Nanodrop) was used to detect the quality and concentration of RNA. Total RNA (400 ng) was reversed transcribed using the PrimeScript RT Reagent kit (Takara, Dalian, China). PCR was performed using the SYBR^®^ Premix Ex Taq™ II (Tli RNaseH Plus) kit (Takara, Japan), according to the manufacturer’s instructions, using an ABI Prism^®^ 7900 Sequence Detection System (Applied Biosystems, Foster City, CA, USA). *U6* and *GAPDH* were used as internal references. Relative expression was calculated using the 2^−ΔΔCt^ method. Primer sequences (Huada, Wuhan, China) are shown in Table S2.

### Protein expression determined using western blot

Western blotting was performed using ccRCC tissues and 786O cells according to previously published protocols [14]. GADPH was used as an internal reference, and the gray intensity of each protein band was analyzed using Image J software.

### Dual-luciferase reporter gene assay

3’UTR-KDM3A-wild type (WT: GGAAAUGAAUUACAGGCAGCUG) and 3’UTR-KDM3A-mutant (MUT: GGAAAUGAAUUACAGGCGGCCG) were cloned downstream of the luciferase gene at the pGL3-luciferase reporter plasmid (Promega, Madison, WI, USA). The constructed vector was identified via sequencing. Cells were seeded into 96-well plates at 70% confluence. After 6 h, luciferase reporter plasmids and RNA were transfected. Six replicates were set up for each sample. DNA and transfection reagents were prepared at Firefly: Renilla: transfection reagent = 0.1 μg: 0.01 μg: 0.25 μl. The final RNA concentration was 100 nM for miRNA and transfection reagent (0.25 μL/well). Diluted DNA, RNA, and transfection reagent were incubated at room temperature for 5 min. Diluted DNA or RNA was then mixed with the transfection reagent and incubated at room temperature for 20 min. Culture medium (50 µl/well) was discarded. DNA and RNA transfection mixtures (25 μL each) were added to each sample. After transfection for 6 h, the medium was replaced with a fresh complete medium. After transfection for a total of 48 h, the medium was discarded. Cells were washed with 100 μl PBS. The remaining PBS was blotted when the 96-well plate was tilted. Diluted 1× protein lysis buffer (50 μl) was added to each well and shaken for 15 min at room temperature. The lysed mixture (10 μl) was added to each well in a white opaque 96-well microtiter plate. Premixed LARII (100 μl) was added. Luciferase intensity was measured after 2 s. Premixed Stop&Glo Reagent (100 μl) was then added to each well and luciferase intensity was measured again after 2 s. Renilla luciferase was used as a reference. The degree of activation of the target reporter gene was compared based on the ratio of the firefly divided by Renilla luciferase.

### Chromatin immunoprecipitation (ChIP)

ChIP assays were performed as described in our previous work [15] using a Pierce Agarose ChIP Kit (Thermo Fisher Scientific). The antibodies used for the ChIP assays were specific for KDM3A (ab91252, Abcam). ChIP-DNA analysis was then performed using qRT-PCR to evaluate the level of YAP1 at the target loci. YAP1 promoter primers are shown in Table S3.

### Cell proliferation was determined using the CCK-8 assay

Cell suspensions (100 µl) were seeded into 96-well plates and incubated in a 37 °C incubator for 2–4 h. CCK-8 (100 µl, Glpbio, Montclair, CA, USA) was added and then incubated for 1–4 h. The absorbance was measured at 450 nm with a reference wavelength of 600–650 nm.

### Cell migration was determined using the scratch test

After cell transfection at 37 °C with 5% CO_2_ for 24 h, a 10 μL pipette tip was used to make one scratch and draw lines on the monolayer cells. Cells were gently washed three times with PBS, serum-free medium was added, and incubated at 37 °C with 5% CO_2_. Images of the cells were taken at 0, 24, and 48 h. Images were taken at distances between the cells on both sides of the scratch and were measured in three randomly selected areas of interest. Scratch distance (%) = (24 h or 48 h scratch distance/0 h scratch distance).

### Cell invasion was determined using the transwell assay

Transwell chambers (8 mm pore size; Corning, New York, USA) were used to perform in vitro cell invasion assays in 24-well plates. In a transwell chamber with a polycarbonate membrane containing matrigel, 600 mL of 20% FBS DMEM medium was pre-added in the lower chamber and equilibrated at 37 °C for 1 h. Cells were resuspended in DMEM medium without FBS 48 h after transfection, inoculated at 1 × 10^6^ cells/mL into the upper chamber, and incubated at 37 °C with 5% CO_2_ for 24 h. The transwell chamber was removed and washed twice with PBS. Cells were fixed with glutaraldehyde and stained with 0.1% crystal violet at 4 °C for 5 min. Cells were then rinsed with PBS twice, surface cells were wiped with a cotton ball and observed under an inverted fluorescence microscope (TE2000, Nikon, Tokyo, Japan). Images were taken in five randomly selected fields. The number of cells passing through the chamber was counted and averaged.

### Immunohistochemistry

Cancer tissues from different groups were fixed in 4% paraformaldehyde, dehydrated using an alcohol gradient, and embedded in paraffin. Tissues were cut to 4 μm serial sections, dewaxed, and rehydrated. Antigen retrieval was performed by microwave heating to remove endogenous peroxidase. After blocking with 10% goat serum for 30 min at room temperature, the primary antibody (50–100 µl) was added and incubated at 4 °C overnight in a wet box. Tissues were then incubated with secondary antibody for 1 h, followed by the addition of 50–100 µl ABC-kit/Streptavidin-HRP and incubated for 30 min at room temperature. After washing, 50–100 µl of freshly prepared DAB (Maxim, Fuzhou, China) was added and incubated at room temperature for ~1 min until a yellow color was observed. The reaction was stopped by immediately washing with water. Tissues were sealed with neutral resin and imaged under a microscope.

### Statistical analysis

All data were analyzed using GraphPad Prism software (GraphPad Software, CA, USA). Data are expressed as mean ± standard deviation. Data comparison between two groups was performed using the paired or unpaired *t* test, where appropriate. Data comparison among multiple groups was performed using one-way ANOVA and Tukey’s post hoc tests. Data comparison between groups at different time points was performed using repeated measures ANOVA and post hoc Bonferroni tests. Correlation between the two groups was tested using Pearson’s correlation coefficient. Calculation data are expressed as rates or percentages and compared using chi-square or Fisher’s exact test. *P < 0.05, **P < 0.01, and ***P < 0.001 were considered statistically significant.

## Results

### miR-335 is downregulated in ccRCC and inhibits tumor growth and metastatic capacity

**Fig. 1:**
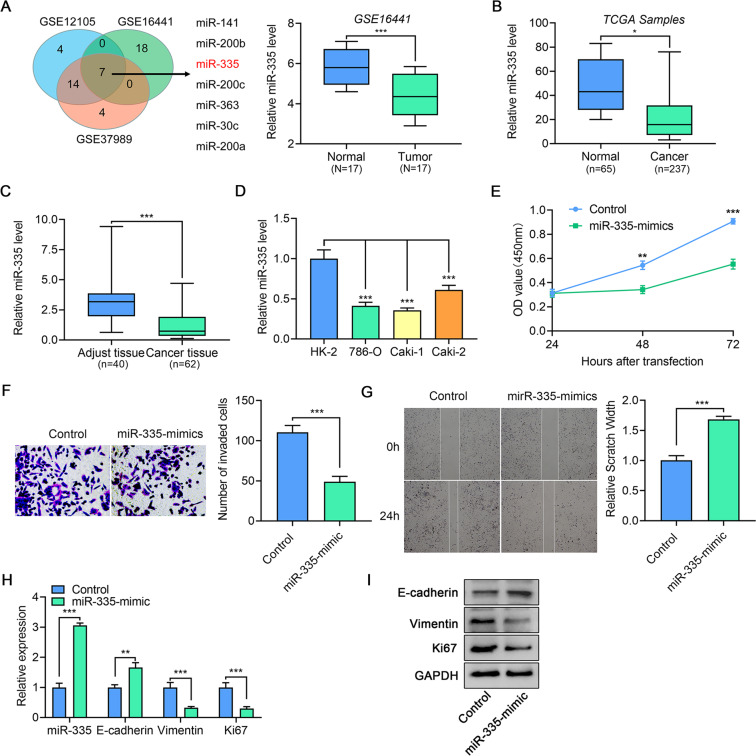
miR-335 is downregulated in ccRCC and inhibited tumor growth and metastatic capacity. **A** Venn analysis on the first seven downregulated miRNAs in three datasets, and miR-335 expression in ccRCC in dataset GSE16441. **B** miR-335 expression in ccRCC in TGCA. **C** miR-335 expression in ccRCC cancer-adjacent normal tissues and cancer tissues. **D** miR-335 expression in ccRCC cell lines (HK2 cells were used as normal control). **E** 786-O cell proliferation was determined using the CCK-8 assay after transfection with miR-335 mimics. **F** 786-O cell invasion was determined using the transwell assay after transfection with miR-335 mimics (200×). **G** 786-O cell migration was determined using the scratch test after transfection with miR-335 mimics. **H**, **I** Ki67, E-cadherin and Vimentin mRNA and protein expression in 786-O cells after transfection with miR-335 mimics. Data are expressed as mean ± standard deviation. Data comparison between two groups was performed using the paired or unpaired *t* test, where appropriate. Data comparison among multiple groups was performed using one-way ANOVA and Tukey’s post hoc test.

To explore novel cancer-related miRNAs in ccRCC, we performed a bioinformatics analysis based on three published miRNA microarray datasets accessed from GEO. As shown in Fig. 1A, a total of seven miRNAs were screened out, showing consistently altered expression in all three datasets. Among them, miR-335 displayed the most varied expression, while also being significantly downregulated in TGCA samples (Fig. 1B). This observation was further confirmed in an independent patient cohort (Fig. 1C). Moreover, the expression of miR-335 was dramatically decreased in patients with higher Fuhrman histological grades and T stages (Table S1). Further, miR-355 levels were markedly lower in primary renal clear cell carcinoma cells 786-O and Caki-2 (However, recent studies have shown that Caki-2 might have the characteristic of papillary RCC [16]) and cutaneous metastatic renal clear carcinoma cells Caki-1 than that in normal proximal tubular cells (HK-2) (Fig. 1D). In the follow-up experiments, we use 786-O cells as the ccRCC model in vitro, for which is a suitable transfection host. These findings suggest that miR-335 may function as an anti-onco-miRNA in ccRCC.

To further investigate the role of miR-335 in ccRCC tumorigenesis, we overexpressed miR-335 using the miRNA mimics in 786-O cells. The CCK-8 assay indicated that miR-335 dramatically blocked the cell growth capacity (Fig. 1E), and the proliferative maker Ki67 downregulated both in mRNA level (Fig. 1H) and protein level (Fig. 1I) in the miR-335 mimic-transfected 786-O cells. Moreover, we also tested whether miR-335 would affect cell invasion and migration capacity using Transwell and wound healing assays. Our results show that both invasion (Fig. 1F) and migration (Fig. 1G) were attenuated in the miR-335 mimic-transfected 786-O cells, and the qPCR (Fig. 1H)and western blot results (Fig. 1I) of E-cadherin and Vimentin also show that miR-335 inhibits tumor metastatic capacity. Together, our studies conclude that miR-335 functions as an important anti-onco-microRNA in ccRCC. Downregulation of miR-335 inhibits tumor growth and metastasis, which prompts tumor progression.

### KDM3A is a downstream target of miR-335, and is overexpressed in ccRCC

**Fig. 2:**
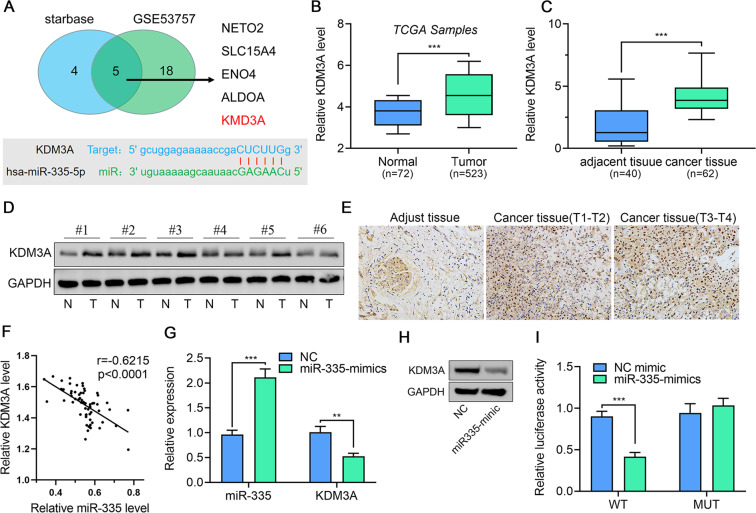
*KDM3A* is a target gene of miR-335 and is overexpressed in ccRCC tumor tissues. **A** Downstream target genes of miR-335 predicted by starBase and GSE53757 dataset. **B** Analysis of KDM3A mRNA expression in ccRCC via TGCA samples. **C**–**E** KDM3A mRNA and protein expression in ccRCC cancer-adjacent normal tissues and cancer tissues determined using qPCR, western blot, and immunohistochemistry (200×). **F** Correlation between miR-335 and KDM3A mRNA expression in ccRCC cancer tissues. **G**, **H** KDM3A mRNA and protein expression in 786-O cells after transfection with miR-335 mimics. **I** Binding relationship between miR-355 and KDM3A determined using the dual-luciferase reporter gene assay. Data are expressed as mean ± standard deviation. Data comparison between two groups was performed using the paired or unpaired *t* test, where appropriate. Correlation between the two groups was tested using Pearson’s correlation.

The downstream target is very important to understand the pathogenic mechanism of miR-335 in ccRCC. We sought targets associated with ccRCC progression, which showed a negative correlation with miR-335. Therefore, we first predicted the downstream target of ccRCC using starBase and identified 1800 targeted mRNAs. Next, these candidates were explored in a gene array dataset that included 144 paired carcinoma and normal ccRCC tissues. Thereafter, we identified five potential target genes, *NETO2*, *SLC15A4*, *ENO2*, *ALDOA*, and *KDM3A*. Among them, histone demethylase KDM3A is an important epigenetic modifier that is reportedly involved in tumor progression in multiple cancers, while miR-335 has a single, highly conserved, predicted target site with the 3-UTR of KDM3A (Fig. 2A). Moreover, we accessed the KIRC TCGA RNAseq dataset and confirmed that the expression of KDM3A was significantly higher in cancer tissues than in cancer-adjacent normal tissues (Fig. 2B). We observed consistent results in our patient cohort. Both mRNA and protein levels of KDM3A were upregulated in cancer tissues, determined using qPCR (Fig. 2C), western blot (Fig. 2D), and IHC (Fig. 2E). Importantly, a negative correlation was observed between miR-335 and KDM3A in ccRCC tissue samples (Fig. 2F). Based on these findings, we selected KDM3A as the downstream target of miR-335 for further study.

To verify the regulation between KDM3A and miR-335, we transfected miR-335 mimics into 786-O cells and detected the expression of KDM3A. As shown in Fig. 2G, miR-335 overexpression resulted in a 50% decrease in KDM3A levels. This downregulation was also observed in KDM3A protein levels (Fig. 2H). In addition, using a dual-luciferase reporter system, we confirmed that the KDM3A decrease occurs in response to regulation by miR-335 (Fig. 2I). Overall, our results conclude that KDM3A is a downstream target of miR-335, and that downregulation of miR-335 contributes to the induction of KDM3A levels in ccRCC.

### KDM3A promotes tumor growth and metastasis in ccRCC

**Fig. 3:**
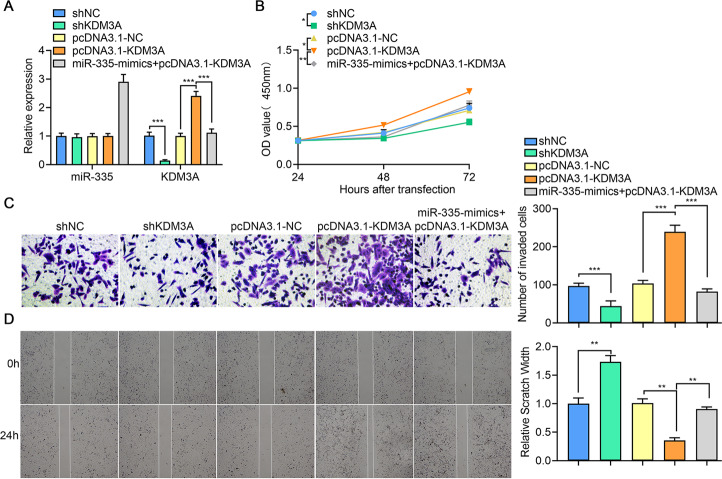
KDM3A promotes tumor growth and metastasis in ccRCC. **A** *miR-335* and *KDM3A* mRNA expression tested using qPCR in different groups of 786-O cells. **B** 786-O cell proliferation determined using the CCK-8 assay in different groups. **C** 786-O cell invasion determined using the transwell assay in different groups (200×). **D** 786-O cell migration determined using the scratch test in different groups. Data are expressed as mean ± standard deviation. Data comparison between two groups was performed using the paired or unpaired *t* test, where appropriate. Data comparison among multiple groups was performed using one-way ANOVA and Tukey’s post hoc test. Data comparison between groups at different time points was performed using repeated measures ANOVA and Bonferroni post hoc test.

As a direct downstream target, KDM3A may potentially contribute to cancer growth and metastasis, due to regulation by miR-335. Thus, to explore the function of KDM3A, we knocked down the expression of KDM3A using shRNA in both control 786-O cells and miR-335 mimic-transfected 786-O cells. As expected, miR-335 decreased KDM3A expression, and the loss of KDM3A did not influence miR-335 expression (Fig. 3A). The CCK-8 assay demonstrated that cell proliferation was significantly reduced in KDM3A shRNA-transfected cells, while increased in KDM3A overexpressed cells. In line with our previous results, miR-335 could inhibit cell proliferation in 786-O cells, and the inhibitory effect of miR-335 was reversed after KDM3A knockdown (Fig. 3B).

In addition, we investigated whether KDM3A contributed to ccRCC metastasis. As shown in Fig. 2C, D, our results showed that a KDM3A deficiency may dramatically attenuate both invasion and migration of ccRCC cells, which was also found in miR-335 overexpressed cells. Overexpression of KDM3A could promote cell invasion and migration. In line with proliferation, the effect of miR-335 or KDM3A was reversed when simultaneously co-transfected with miR-335 mimics and KDM3A overexpression plasmids. Taken together, these findings indicate that KDM3A is an important oncogene in ccRCC, and promotes tumor growth and metastasis while functioning as the main executor of miR-335.

### KDM3A epigenetically activates YAP1 expression

**Fig. 4:**
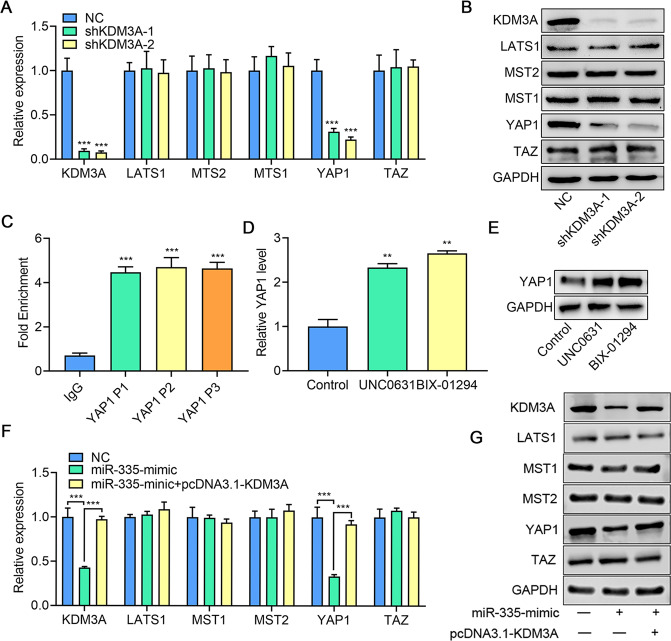
KDM3A up-regulates YAP1 expression through demethylation in 786-O cells. **A**, **B** mRNA and protein expression of Hippo pathway molecules after KDM3A knockdown with shRNAs. **C** Enrichment of KDM3A demethylase on the YAP1 promoter detected using ChIP-qPCR. **D**, **E** YAP1 mRNA and protein expression after treatment with H3K9m2 inhibitors. **F** mRNA and protein expression of Hippo pathway molecules after miR-335 and KDM3A overexpression. Data are expressed as mean ± standard deviation. Data comparison between two groups was performed using paired or unpaired *t* test, where appropriate. Data comparison among multiple groups was performed using one-way ANOVA and Tukey’s post hoc test.

KDM3A is an H3K9me1/2 histone demethylase. Due to the methylation of H3K9 in promoter regions, which inhibits transcription, KDM3A demethylase activity tends to enhance gene expression. YAP1 is a transcriptional regulator shown to promote tumorigenesis in multiple cancers [17]. In ccRCC, YAP inhibition reportedly impairs LIFR-silencing promotion of cell migration and invasion [18]. Importantly, KDM3A has been shown to upregulate YAP1 and the Hippo pathway in colorectal cancer [19]. Thus, to further address the mechanisms by which KDM3A promotes the oncogenic phenotype in ccRCC, we first investigated gene alteration in the Hippo pathway after KDM3A knockdown in 786-O cells. Results show that KDM3A shRNA transfection reduced the mRNA expression of *YAP1* (Fig. 4A). This observation was confirmed at the protein level using western blot (Fig. 4B), suggesting that YAP1 is a downstream target of KDM3A. In addition, the direct interaction between KDM3A and YAP1 was identified using ChIP-PCR (Fig. 4C). We identified the robust binding of KDM3A to the YAP1 promoter. Moreover, using histone methyltransferase inhibitors, BIX-01294, and UNC0631, we observed significant YAP1 increases at both mRNA (Fig. 4D) and protein (Fig. 4E) levels. Except KDM3A, the expression of YAP1 may be regulated by miR-335 and reversed after KDM3A overexpression, as observed using qPCR (Fig. 4F) and western blot (Fig. 4G), suggesting a closed relationship between miR-335, KDM3A, and YAP1. Overall, our findings conclude that YAP1 is the direct target of KDM3A in ccRCC, and KDM3A may regulate YAP1 expression.

### The miR-335/KDM3A/YAP1 axis is the key regulator of tumor growth and metastasis in ccRCC

**Fig. 5:**
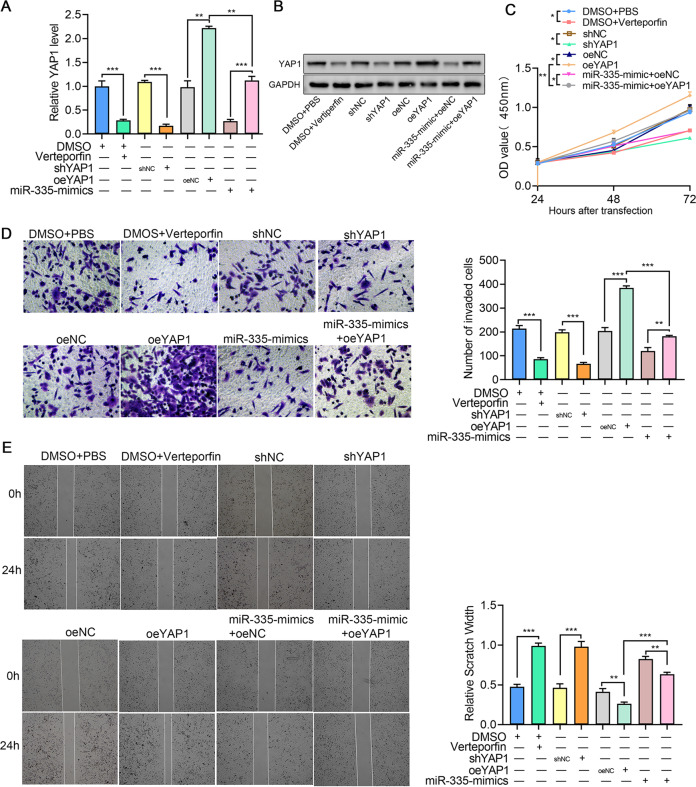
miR-335 inhibits 786-O cell proliferation, migration, and invasion via the KDM3A/YAP1 axis. **A**, **B** YAP1 mRNA and protein expression in 786-O cells with various transfections. **E** 786-O cell proliferation determined using the CCK-8 assay. **F** 786-O cell invasion determined using the transwell assay (200×). **G** 786-O cell migration determined using the scratch test. Data are expressed as mean ± standard deviation. Data comparison among multiple groups was performed using one-way ANOVA and Tukey’s post hoc test. Data comparison between groups at different time points was performed using repeated measures ANOVA and Bonferroni post hoc test.

Our studies have established a regulatory axis including miR-335, KDM3A, and YAP1. Thus, we performed a co-transfection assay to further explore whether YAP1 is also involved in miR-335-induced inhibition of tumor growth and metastasis, as is KDM3A. First, the specific inhibitor, shRNA, and overexpression plasmids were treated or transfected into 786-O cells, respectively. Then, the CCK-8 assay revealed that the inhibitor and shRNA suppressed proliferation, whereas overexpression plasmids promoted 786-O cell proliferation (Fig. 5C). Importantly, after simultaneously co-transfecting YAP1 and miR-335 into 786-O cells, the molecular effects were reversed by opposing treatments. In addition, invasion (Fig. 5D) and migration (Fig. 5E) abilities were also inhibited by the YAP1 inhibitor or shRNA, and promoted by YAP1 overexpression, while the co-transfection of YAP1 and miR-335 showed modest alterations, compared to the single transfection group. Together, our findings verified the functional correlation between miR-335 and YAP1, and identified a new miR-335/KDM3A/YAP1 regulatory axis, which plays an important role in tumor growth and metastasis in ccRCC.

## Discussion

**Fig. 6:**
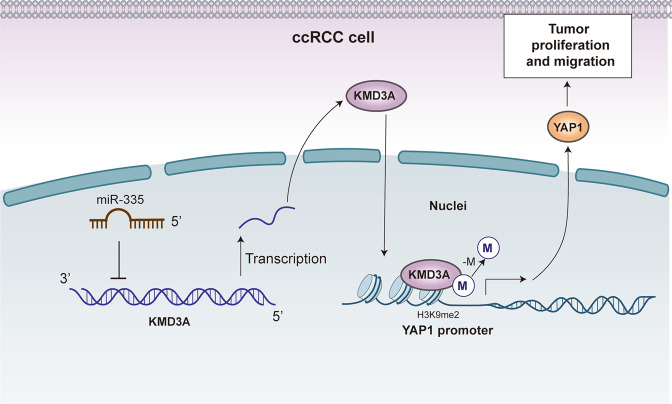
Schematic diagram showing the involvement of miR-335 in ccRCC. miR-355 is under-expressed in ccRCC. Increased KDM3A expression promotes the demethylation of the YAP1 promoter. Increased YAP1 expression (in the Hippo pathway) promotes ccRCC cell proliferation, migration, and invasion.

Epigenetic regulatory mechanisms, involving DNA methylation, histone modification at the genomic transcriptional level, and the interaction of non-coding RNAs, in particular miRNAs, with mRNA at the post-transcriptional level, are important in the regulation of genes and proteins [20]. Alterations in the expression or regulatory function of miRNAs are key factors for pathological processes. To date, over 300 diseases have been correlated with miRNAs, including oncological diseases [21]. miRNAs may regulate all characteristics of tumors, including control of cell proliferation, apoptosis, angiogenesis, tissue invasion, and metastasis. Here, we focused on miR-335, the most downregulated miRNA observed in three different miRNA array datasets of ccRCC. Functional assays revealed that overexpression of miR-335 inhibits cell proliferation, migration, and invasion in ccRCC cell lines. Moreover, we found H3K9me1/2 histone demethylase KDM3A as a novel downstream target of miR-335, and identified a new miR-335/KDM3A/YAP1 regulatory axis, which appears to play important roles in ccRCC tumor growth and metastasis. Overall, our studies report a new epigenetic-based tumor-promotional pathway in ccRCC and provide a potential target for the treatment of metastasized ccRCC (Fig. 6).

miRNAs are classified as oncogenic miRNAs or anti-onco-microRNAs (tumor suppressive) based on their stimulatory or inhibitory effects, respectively, on tumor development. The expression of miR‐335 has been shown to be aberrant in multiple cancers. Decreased expression of miR-335 was reported in gastric cancer [22], colorectal cancer [23], and non‐small cell lung cancer [24]. However, increased expression of miR-335 was found in several hematologic cancers, such as multiple myeloma [25] and acute leukemia [26]. It is believed that miR-335 can be oncogenic or onco-suppressive depending on the type of cancer. Based on our research, miR-335 was downregulated in ccRCC tumor tissue compared with cancer-adjacent normal tissues, and the expression of miR-335 negatively correlated with histological classification and tumor stage of ccRCC. We also observed a tumor-suppressive role in miR-335 mimic-transfected cells. Thus, miR-335 represents an important tumor-suppressive mechanism in ccRCC. In line with our results, Wang et al. reported the decreased expression of miR-335 in a relatively small patient cohort. Their results showed that the low expression of miR-335 was associated with lymph node metastasis, larger tumor size, and poor T stage [27]. Thus, due to its expression profile and tumor suppressor function, miR-335 may also be used as a metastasis predictive marker for ccRCC [28, 29].

In the present study, to identify a direct target of miR-335, we combined miRNA-target interaction predictor, starBase [30], and a ccRCC gene array dataset. Among all candidates, KDM3A was found to be upregulated in ccRCC, and negatively correlated with the expression of miR-335. Moreover, the direct interaction between miR-335 and KDM3A was identified using luciferase assays. KDM3A is a histone modifier that catalyzes the demethylation of transcriptionally repressive mono- and di-methylated histone H3 lysine 9 (H3K9me1/me2), thereby mediating transcriptional activation. The deregulation of KDM3A has been reported in skin, hair, and cardiovascular diseases, as well as in multiple cancers, including breast, prostate, and colon cancers [31]. The function of KDM3A is differential and complicated in both physiological and pathological processes. Unlike miR-335, KDM3A is frequently upregulated in cancers and elevated KDM3A correlates with poor disease prognosis [32, 33]. Deletion of KDM3A was found to inhibit colony formation [34], cell proliferation [35], migration [36], and invasion [37]. Thus, KDM3A is an important regulator for tumor growth and metastasis. Here, we explored the pathogenesis of KDM3A in ccRCC. Our results showed that knockdown or overexpression of KDM3A may respectively inhibit or promote cell proliferation, invasion, and migration. Thus, we show that KDM3A is not only a direct downstream target of miR-335, but also a compelling drug target due to its regulatory roles in cancer progression.

Hippo signaling exerts a critical role in modulating cell proliferation and has been demonstrated as an important oncogenic pathway that contributes to the progression of cancer [38]. The Hippo signaling pathway is primarily composed of MST1/2, LATS1/2, YAP, and/or its paralog TAZ [39]. Among them, YAP is the transcriptional co-activator protein that participates in the regulation of downstream genes [40]. The overexpression or over activation of YAP is a common phenomenon in cancers, including non-small cell lung cancer [41], hepatocellular cancer [42], colon cancer [43], gastric cancer [44], and ccRCC [45]. In our study, we showed that the expression of YAP may be regulated by KDM3A directly through an epigenetic role. The binding site of KDM3A was identified in the promoter region of YAP1. Moreover, KDM3A knockdown dramatically influences the intracellular level of YAP1, while using a histone methylation inhibitor would upregulate YAP1 expression. Our findings established a miR-335/KDM3A/YAP1 regulatory axis in ccRCC. Our experiments have shown that the inhibitory role of miR-335 in cell proliferation, invasion, and migration relied on both KDM3A and YAP1, supporting the essential role of the miR-335/KDM3A/YAP1 axis in ccRCC tumor growth and metastasis.

In summary, we screened and identified a new pathogenesis-related miRNA, miR-335, and its downstream regulation axis. Our study lays a foundation for further understanding of the molecular mechanisms of ccRCC progression and indicates that miR-335 and KDM3A are important targets to treat metastasized ccRCC.

## Supplementary information

original western blot results

Original qPCR

Table S1

Table S2

Table S3
