# Supplementary material for: Downregulation of miR-335 exhibited an oncogenic effect via promoting KDM3A/YAP1 networks in clear cell renal cell carcinoma
Source: Cancer Gene Ther. 2021 Apr 23;29(5):573–84. doi: 10.1038/s41417-021-00335-3 (PMC9113937; doi:10.1038/s41417-021-00335-3)
Supplement: Supplementary file 3 — Table S1 [file 41417_2021_335_MOESM3_ESM.docx]

Table S1.Correlations between miR-335 and KDM3A expression and clinicopathologic features in ccRCC patients

| Patient characteristics  (n=62) | miR-335 qPCR results | | | KDM3A IHC results | | |
| --- | --- | --- | --- | --- | --- | --- |
|  | Low  (<0.552) | High  (≥0.552) | 1. Value   (high vs. low)^a^ | Low  (<0.50) | High  (≥0.50) | *P*-Value  (high vs. low)^a^ |
| Age (years)  Mean: 55.95  Range: 30-81 |  | | |  | | |
| ≤56 | 15 | 16 | <0.7991 | 15 | 16 | <0.3588 |
| ＞56 | 14 | 17 |  | 12 | 19 |  |
| sex |  | | |  | | |
| Female (n=35) | 21 | 16 | <0.0553 | 19 | 18 | <0.7630 |
| Male (n=27) | 8 | 17 |  | 11 | 14 |  |
| Fuhrmans histological grade |  | | |  | | |
| 1+2 (n=25) | 16 | 9 | <0.0255 | 8 | 17 | <0.0059 |
| 3+4 (n=37) | 13 | 24 |  | 11 | 26 |  |
| TNM stage |  | | |  | | |
| T1-T2 | 15 | 7 | <0.0122 | 7 | 15 | <0.0010 |
| T3-T4 | 14 | 26 |  | 10 | 30 |  |
| a *P*-values were calculated by Fisher's 2x2 test. ccRCC, clear cell renal cell carcinoma. | | | | | | |
