# Supplementary material for: Downregulation of miR-335 exhibited an oncogenic effect via promoting KDM3A/YAP1 networks in clear cell renal cell carcinoma
Source: Cancer Gene Ther. 2021 Apr 23;29(5):573–84. doi: 10.1038/s41417-021-00335-3 (PMC9113937; doi:10.1038/s41417-021-00335-3)
Supplement: Supplementary file 4 — Table S2 [file 41417_2021_335_MOESM4_ESM.docx]

Table S2. Primer sequences used for qRT-PCR

| Gene | | Primer |
| --- | --- | --- |
| *GAPDH* | forward | GGAGCGAGATCCCTCCAAAAT |
|  | reverse | GGCTGTTGTCATACTTCTCATGG |
| *U6* | forward | AGTAAGCCCTTGCTGTCAGTG |
|  | reverse | CCTGGGTCTGATAATGCTGGG |
| *miR-335* | forward | GTCGTATCCAGTGCAGGGTCCG |
|  | reverse | GTGCAGGGTCCGACCT |
| *KDM3A* | forward | TTTGGAGTATGTGTGGACTG |
|  | reverse | TTCCAGGAATGATCTGTGTG |
| *TAZ* | forward | CTGATTGCTGAGTGTCATCT |
|  | reverse | CGATCAGCACAGTGATTTTC |
| *MST1* | forward | CTGTGTTTGTAGCCATACGCA |
|  | reverse | GGGCTCTAGGTCATGCAGG |
| *MST2* | forward | AGGAACAGCAACGAGAATTGG |
|  | reverse | CCCCTTCACTCATCGTGCTT |
| *LATS1* | forward | AATTTGGGACGCATCATAAAGCC |
|  | reverse | TCGTCGAGGATCTTGGTAACTC |
| *YAP1* | forward | AGACCACTCCTTGTGAAATCCC |
|  | reverse | GTAGCCTTGAGACCCCATCG |
| Ki67 | forward | TTCGCAAGCGCATAACCCA |
|  | reverse | AACCGTGTCACAGTGCCAAA |
| E-cadherin | forward | CGAGAGCTACACGTTCACGG |
|  | reverse | GGGTGTCGAGGGAAAAATAGG |
| Vimentin | forward | GACGCCATCAACACCGAGTT |
|  | reverse | CTTTGTCGTTGGTTAGCTGGT |
