# Supplementary material for: Downregulation of miR-335 exhibited an oncogenic effect via promoting KDM3A/YAP1 networks in clear cell renal cell carcinoma
Source: Cancer Gene Ther. 2021 Apr 23;29(5):573–84. doi: 10.1038/s41417-021-00335-3 (PMC9113937; doi:10.1038/s41417-021-00335-3)
Supplement: Supplementary file 5 — Table S3 [file 41417_2021_335_MOESM5_ESM.docx]

Table S3. YAP1 promoter primer sequences

| YAP1 | | Primer |
| --- | --- | --- |
| *P1* | forward | CCGTTTACCCCTCTCAAGTG |
|  | reverse | GTGAGCCGGCATTGATGTT |
| *P2* | forward | CGCACATCCTCTCTCCACTT |
|  | reverse | CCTTGGCTGCAGGAAGTTCT |
| *P3* | forward | CGTTTGAGGCGAGTTTCTGT |
|  | reverse | CGCCTCCCCTTTCTCTTTAT |
